# Statin-induced risk of diabetes does not reduce cardiovascular benefits in primary prevention: a 6-year propensity-score matched study in a large population

**DOI:** 10.1186/s12933-025-02798-2

**Published:** 2025-05-31

**Authors:** Maria Lembo, Valentina Trimarco, Raffaele Izzo, Daniela Pacella, Stanislovas S. Jankauskas, Paola Gallo, Roberto Piccinocchi, Carmine Morisco, Gaetano Piccinocchi, Luca Bardi, Stefano Cristiano, Giovanni Esposito, Giuseppe Giugliano, Fahimeh Varzideh, Maria Virginia Manzi, Bruno Trimarco, Gaetano Santulli

**Affiliations:** 1 Department of Advanced Biomedical Sciences, “Federico II” University, Naples, Italy; 2 Department of Neuroscience, Reproductive Sciences, and Dentistry, “Federico II” University, Naples, Italy; 3 Department of Public Health, “Federico II” University, Naples, Italy; 4 Department of Medicine, Einstein-Mount Sinai Diabetes Research Center (ES-DRC), Fleischer Institute for Diabetes and Metabolism (FIDAM), Wilf Family Cardiovascular Research Institute, Albert Einstein College of Medicine, New York City, NY USA; 5 “Luigi Vanvitelli” Hospital, Naples, Italy; 6 Academic Research Unit, International Translational Research and Medical Education (ITME) Consortium, Naples, Italy; 7 Italian Society for Cardiovascular Prevention (SIPREC), Rome, Italy; 8 COMEGEN Primary Care Physician Cooperative, Italian Society of General Medicine (SIMG), Naples, Italy; 9 Department of Molecular Pharmacology, Einstein Institute for Aging Research, Institute for Neuroimmunology and Inflammation (INI), Albert Einstein College of Medicine, New York City, NY USA

## Abstract

**Background:**

The long-term risk of cardiovascular (CV) events in individuals who develop new-onset type 2 diabetes (T2D) after having received statin therapy in primary prevention is mostly unknown.

**Methods:**

We designed a population-based cohort study in individuals without T2D and atherosclerotic CV disease (ASCVD), divided in two groups according to the presence of statin therapy. We also balanced the study groups for demographic and clinical factors using propensity score matching.

**Results:**

119307 individuals without T2D and ASCVD were divided in statin users (*N* = 90906) or not (*N* = 28401) and followed-up for 70.1 ± 61.3 months. Yearly incidence of T2D rate was 0.3% in the control group and 2.2% in the statin treated group. A Cox regression analysis confirmed the association between incident T2D and statin therapy. In normotensive individuals, the presence of statin therapy led to a 2-fold risk to develop incident T2D (HR: 2.61;95% CI: 2.11–3.22, *p* < 0.001). In the hypertensive population, statin therapy was associated with a HR of incident T2D of 4.62 (95% CI: 3.75–5.69, *p* < 0.001). The rate of CV events, including coronary and cerebrovascular fatal and non-fatal events, was 1.9% in the statin group vs. 0.7% in the control group and a multiple regression analysis demonstrated an association between statin therapy and CV events. A further Cox regression performed only in the statin treated population revealed a significant association of CV events with age, serum creatinine levels, and incident T2D. Of note, the increased rate of new-onset T2D associated with statin use does not modify the class of CV risk of this population. All these findings were confirmed by propensity score matched analysis.

**Conclusions:**

Statin therapy in primary prevention is associated with a higher risk of incident T2D, especially in hypertensive patients. However, since the final CV risk of those who develop T2D during statin treatment was lower than the one required for statin prescription according to the ESC guidelines, indicating that this phenomenon does not impair the benefit in CV prevention associated with the lipid lowering effect of statins.

**Supplementary Information:**

The online version contains supplementary material available at 10.1186/s12933-025-02798-2.

## Research insights summary

**What is currently known about this topic?**

- Statins increase the risk to develop incident type 2 diabetes (T2D) in patients with atherosclerotic cardiovascular disease (ASCVD).

**What is the key research question?**

- Is statin therapy in primary prevention associated with increased new-onset T2D? What is the risk of cardiovascular (CV) events in patients developing T2D after receiving statins?

**What is new?**

- Statin therapy in primary prevention is associated with an increased risk of incident T2D, particularly evident in hypertensives.
- Patients with incident T2D assuming statins had an increased risk of CV events, but the risk of CV events remained below the one suggested by guidelines for the prescription of statins.

**How might this study influence clinical practice?**

- The risk of developing T2D after receiving statins in primary prevention should not discourage their use.

## Introduction

Statins have few confirmed adverse effects [1–5] but recent meta-analyses of large randomized controlled trials (RCT) suggest that standard regimens may increase the risk of new-onset type 2 diabetes (T2D) by about 10% compared to placebo or usual care and more intensive statin regimens produced a further 10% relative increase in risk [6–15]. In order to provide insights on which types of patients are at particularly high risk of developing T2D due to statin treatment, the timing of any excess risk after commencing therapy, or the effects of statin therapy on glycemic control in people with diabetes [16–20], the Cholesterol Treatment Trialists’ (CTT) Collaboration [21] sought individual participant data on all recorded diabetes-related adverse events, treatments for diabetes, and measures of glycemia recorded within their large, long-term, double-blind RCTs of statin therapy. They found that statins cause a moderate dose-dependent increase in new diagnoses of T2D that is consistent with a small upwards shift in glycemia, mainly in people with baseline glycemic markers that are close to the diagnostic threshold for diabetes [21].

More recently, Lee and coworkers [22] performed a systematic review and individual patient data meta-analysis from RCTs evaluating the long-term efficacy and safety of an alternative LDL cholesterol–lowering strategy compared with a high-intensity statin strategy in patients with atherosclerotic cardiovascular (CV) disease (ASCVD), and observed that also the alternative strategy group had an increase in the rate of new-onset diabetes, but smaller than with high intensity statins.

However, these analyses primarily included studies involving patients with established ASCVD, making it unclear whether statins also increase the risk of new-onset T2D in the context of primary prevention; it also remains uncertain whether any potential adverse effect of statins on CV risk due to this phenomenon is fully offset by the overall reduction in CV events observed with statin therapy, as suggested in secondary prevention populations [21].

We have previously reported that in a large sample of non-diabetic hypertensive patients, uncontrolled blood pressure is associated with twofold increased risk of incident diabetes independently of age, body mass index (BMI), baseline blood pressure, or fasting plasma glucose [23]. Subsequently, we evaluated the risk of incident T2D in relation to statin prescription in 4750 hypertensive, non-diabetic outpatients from the “*Campania Salute Network*”, without chronic kidney disease more than grade 3, free of prevalent CV disease and with at least 12 months of follow-up and at the end of follow-up, prevalence of T2D was 18.1% among patients on statins and 7.2% among those without lipid lowering therapy (*p* < 0.0001). However, incident T2D was 10.2% in patients on statins and 8.7% in those free of statin therapy, a difference which did not achieve statistical significance.

Hence, we designed the present study to verify in a large real-world population whether statin therapy in primary prevention is independently associated with an increased probability of new-onset T2D, either in absence or in presence of hypertension, and how the increased rate of new T2D, if any, contributes to the occurrence of CV events during a long follow-up period.

## Methods

We conducted a population-based retrospective cohort study using the database of COMEGEN (“COoperativa di MEdicinaGENerale”: General Medicine Cooperative), an organization of primary physicians in the Naples Local Health Authority of the Italian Ministry of Health (“*ASL Napoli 1 Centro*”); as we recently described in a paper published in the *Journal of Clinical Investigation* [24].

The main outcome of the study was the new diagnosis of T2D assessed by the ICD-X codes E11 (“T2D”) and E13X (“Other specified diabetes mellitus”) defined as at least two measurements (not necessarily consecutive) of fasting plasma glucose concentration 126 mg/dl or higher or at least one HbA1c value of 6.5% (48 mmol/mol) or higher, or prescription of antidiabetic therapies for more than 30 days [15]. Diagnoses of T1D, including the specific code E08, were excluded. At the beginning of the observation period, we removed from the study cohort all individuals with a record of HbA1c > 6.5% (48 mmol/mol), or a previous diagnosis of T2D as defined by ICD-X codes E08.X to E13.X, or prescription record of antidiabetic medications for more than 30 days, as well as those with an ASCVD. The secondary outcome of our study was a composite of incident CV events that included fatal and nonfatal myocardial infarction or stroke.

### Study sample

We selected all patients with age between 18 and 90 years with available physical measurements including weight, height, body mass index (BMI), heart rate and blood pressure; biochemical measurements included serum creatinine fasting blood glucose, total-cholesterol, HDL and LDL-cholesterol, triglycerides, serum transaminases, hemoglobin and platelet counts; medical conditions included coronary/carotid events, diabetes, hypertension, and smoking status. Current chronic therapies were categorized into three classes: antihypertensive agents, antidiabetic agents, lipid-lowering drugs.

We excluded patients with liver cirrhosis (which has been linked to non-trivial safety concerns that could discourage the use of statins [25]) and patients with conditions that could limit life expectancy (cancer, peripheral vascular disease, venous thrombosis, abdominal aortic aneurysm, valvular heart disease, and dementia).

The follow-up time was defined as the time from enrollment until the end of follow-up, incident CV event or death, or loss to follow-up, whichever came first. To adjust for the difference in baseline characteristics between groups, we used clinically available predefined variables, which were selected on the basis of a previous report [15], in which diabetic risk score was defined. Predefined baseline variables included age, sex, BMI, smoking status (current, former, or never), total serum cholesterol, fasting plasma glucose, and creatinine. Comorbidities such as hypertension, hypercholesterolemia, and hypertriglyceridemia were also included as predefined covariates.

## CV risk factor and disease assessment

Data on demographics and risk factors, including age, sex, race, history of myocardial infarction, diabetes, hypertension, stroke, and smoking habit, were recorded at enrollment. Hypertension was defined as a record of systolic blood pressure > 140 mmHg and/or diastolic blood pressure > 90 mmHg, or a previous diagnosis of hypertension as defined by the ICD-X (code I-10), or prescription record of anti-hypertensive medications for more than 30 days. Lipid measurements on fasting blood samples were implemented at each examination [19]. Triglycerides and total cholesterol levels were measured enzymatically, high-density lipoprotein cholesterol (HDL-C) was obtained after precipitation with dextran sulfate/magnesium chloride, and LDL-C was calculated applying the Friedewald equation, as reported earlier [26].

We stratified the study population into two groups based on the presence of statin therapy. The following statins were administered alone or in combination with ezetimibe: atorvastatin, fluvastatin, lovastatin, rosuvastatin, simvastatin, and pravastatin, with the dosage adjusted during the follow-up if necessary, according to efficacy and tolerability. Adherence to pharmacological treatment was assessed by the number of prescriptions, inasmuch as these types of drugs are furnished for free by the National Health Service; physicians asked patients about drug compliance and adverse reactions to confirm drug exposure and tolerability at each visit.

### Statistical analysis

Data were analyzed using IBM SPSS (version 29) and R statistical software version 4.4.0. Continuous variables are expressed as mean ± standard deviation, while categorical variables as relative frequencies. Differences are expressed as standardized mean difference (SMD) or as two-sample test for equality of proportions (with continuity correction) as appropriate. Cumulative Hazard functions for incident T2D and CV events were estimated using adjusted Cox regression analysis comparing participants taking vs. not taking statins, including all covariates that significantly differed between the subgroups, i.e. age, sex, BMI, fasting plasma glucose, serum creatinine, total serum cholesterol, hypertension and follow-up time. The proportional hazard assumption was tested using the Schoenfeld residuals. Sensitivity analyses included removing individuals with missing data or substituting outliers with missing values. Then, we run separate adjusted Cox model with T2D as outcome adding the interactions for the combinations of categorical variables with statins, and in particular with sex and age (categorized as < 40, 40–65, > 65).

In order to confirm the results obtained at this exploratory analysis, given that imputation was not possible due to the large proportion of missing values only on selected variables (BMI, fasting plasma glucose, creatinine and cholesterol), to prevent introducing biases, a propensity score model was implemented, as we previously described [27, 28], to achieve matching of the statin treated and non-treated groups by the same aforementioned covariates. The propensity score matching model was run on cases without any missing values (complete cases) for the mentioned variables. 16446 participants had complete data available (of which 8502 on statin treatment). Optimal matching was achieved using the nearest neighbor matching, with a 1:1 ratio without replacement (greedy algorithm) with a caliper equal to 0.1. After matching, all SMDs for the covariates were below 0.1. Unadjusted Cox regression models were then used to estimate cumulative hazard curves of T2D and CV events on the two statin-treatment matched cohorts. A two-tailed p-value < 0.05 was considered statistically significant.

## Results

### Study participants

**Fig. 1:**
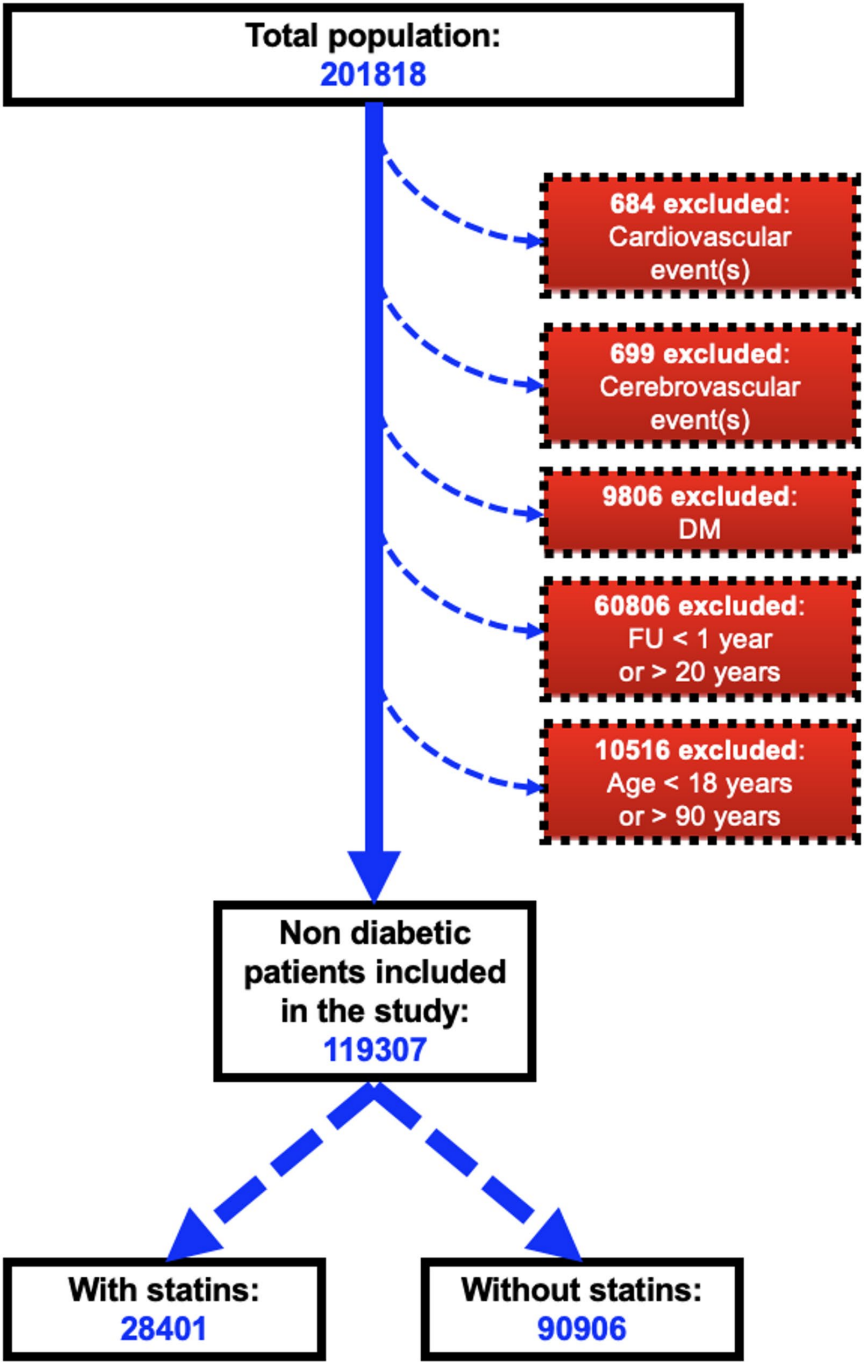
Flow chart of the study showing the selection of the study population

Our total sample included 201818 individuals, among these 11189 were excluded for prevalent T2D or CV diseases, and 60806 on account of a follow-up period < 1 or > 20 years. Among the remaining 129823 patients, 10532 satisfied the exclusion criteria. Thus, our study population included 119307 non-diabetic individuals with no history of ASCVD (Fig. 1), with a mean follow-up duration of 70.1 ± 61.3 months.

The baseline demographic characteristics of this cohort, stratified by presence (*N* = 28401) or absence (*N* = 90906) of statin therapy are summarized in Table 1. Compared to control individuals, those treated with statins were older, were less likely to be male, were more likely to have higher values of BMI, fasting plasma glucose and serum creatinine and total cholesterol. Also the probability to have arterial hypertension was greater, while smoking habit and serum triglyceride levels were comparable between the two study groups. Finally, the mean persistence to statin therapy as assessed by requests of prescription was 90%, while mean self-reported drug compliance was above 80%.

**Table 1:** Baseline demographic characteristics of the study cohort divided in two groups according to Statin therapy

	No statins (*N* = 90906)	Statins (*N* = 28401)	Difference (95% CI)
Age (years)	44.3 ± 16.7	66.0 ± 12.3	-1.4 (-1.4, -1.4)
Sex (M/F)	46.6/53.4	44.4/55.6	0.05 (0.03, 0.06)
*Missing*	*16*	*0*	
BMI (Kg/m^2^)	25.3 ± 4.6	26.8 ± 4.1	-0.34 (-0.36, -0.32)
*Missing*	*67*,*606*	*13*,*154*	
Fasting plasma glucose (mg/dl)	90.4 ± 14.4	97.9 ± 17.7	-0.48 (-0.50, -0.45)
*Missing*	*73*,*499*	*15*,*024*	
Serum creatinine (mg/dl)	0.85 ± 0.2	0.91 ± 0.3	-0.31 (-0.33, -0.29)
*Missing*	*73*,*287*	*14*,*700*	
Hypertension (%)	22.8	78.1	-0.50 (-0,51, -0.50)
Follow-up (months)	69.7 ± 61.0	71.2 ± 62.3	-0.02 (-0.04, -0.01)
Total cholesterol (mg/dl)	185.1 ± 35.7	191.4 ± 47.5	-0.15 (-0.17, -0.13)
*Missing*	*71*,*391*	*11*,*420*	
LDL cholesterol (mg/dl)	111 ± 30	116 ± 37	-0.16 (-0.19, -0.13)
*Missing*	*82*,*340*	*20*,*023*	
Triglycerides (mg/dl)	96 ± 43	121 ± 47	-0.56 (-0.59, -0.53)
*Missing*	*82*,*236*	*19*,*983*	

### T2D diagnosis

The absolute numbers of new diagnoses of T2D during the follow-up were 1475 and 3448 in the control and in the statin treated group, respectively. T2D incidence rate was 1.6% in the control group and 12.1% in the statin treatment group (*p* < 0.001). Since major determinants of T2D development were prevalent in the group of patients treated with statins, a multiple regression analysis was performed taking into account all those possible determinants, including age, sex, fasting plasma glucose, BMI, serum creatinine, total serum cholesterol, statin therapy and hypertension. Smoking habits and serum triglycerides levels were excluded from Cox regression since they did not differ between the two groups.

**Fig. 2:**
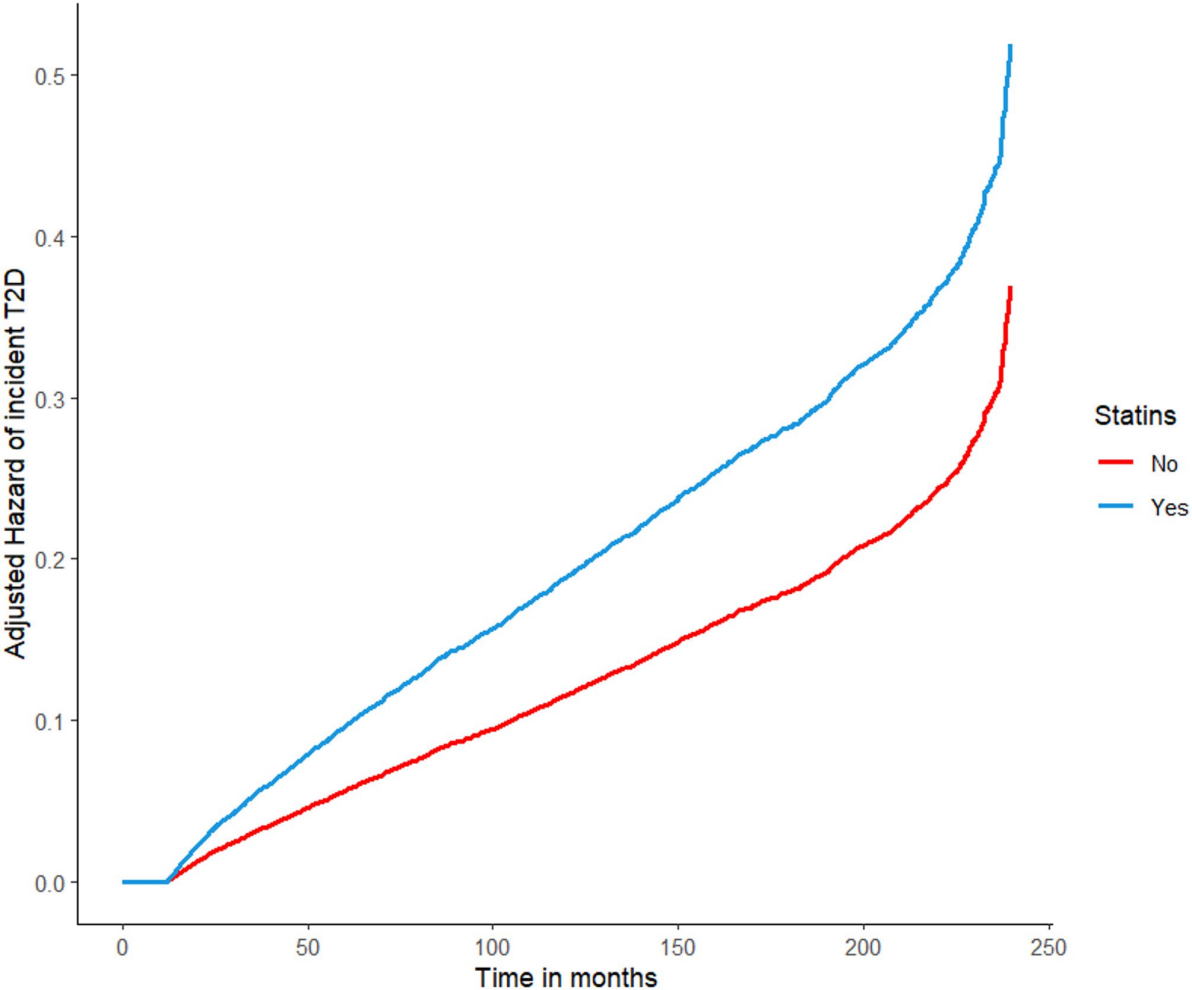
Adjusted hazard of incident T2D during the follow-up by statin therapy

The results of the regression analysis performed to investigate the variables that are associated with the risk of T2D over the observation period are reported in Table 2. The results of the adjusted model are computed on complete cases (*n* = 16446). All the considered predictive factors, except gender, were significantly associated with the risk to develop T2D. However, the Cox regression showed that the time course of the hazard of incident T2D during the follow-up was different (*p* < 0.001) between the two study groups. In particular, as showed in Fig. 2 the two curves of hazard incident of T2D start to diverge about after one year of statin therapy and then the risk of incident T2D progressively increases in patients treated with statins, reaching at the end of the follow-up a 7.5-fold higher incidence of T2D in the statin group compared to the control group. Results on separate models showed no significant interaction (considering as reference class age < 40, statin*40–65 *p* = 0.900, statin*>65 *p* = 0.684, statin*sex male *p* = 0.389).

**Table 2:** Multiple Cox regression analysis performed to investigate variables associated with the risk of incident T2D over the observation period

Outcome incident T2D	aHR	95% CI	*p*-value
Age (years)	1.02	1.02, 1.03	< 0.001
Sex F vs. M	0.98	0.90, 1.07	0.670
Fasting plasma glucose (mg/dl)	1.03	1.03, 1.03	< 0.001
Statin therapy	1.81	1.64, 1.99	< 0.001
BMI (kg/m^2^)	1.04	1.03, 1.05	< 0.001
Serum creatinine (mg/dl)	1.37	1.17, 1.61	< 0.001
Hypertension	1.67	1.48, 1.88	< 0.001
Total cholesterol (mg/dl)	0.99	0.99, 0.99	< 0.001

### Arterial hypertension

**Fig. 3:**
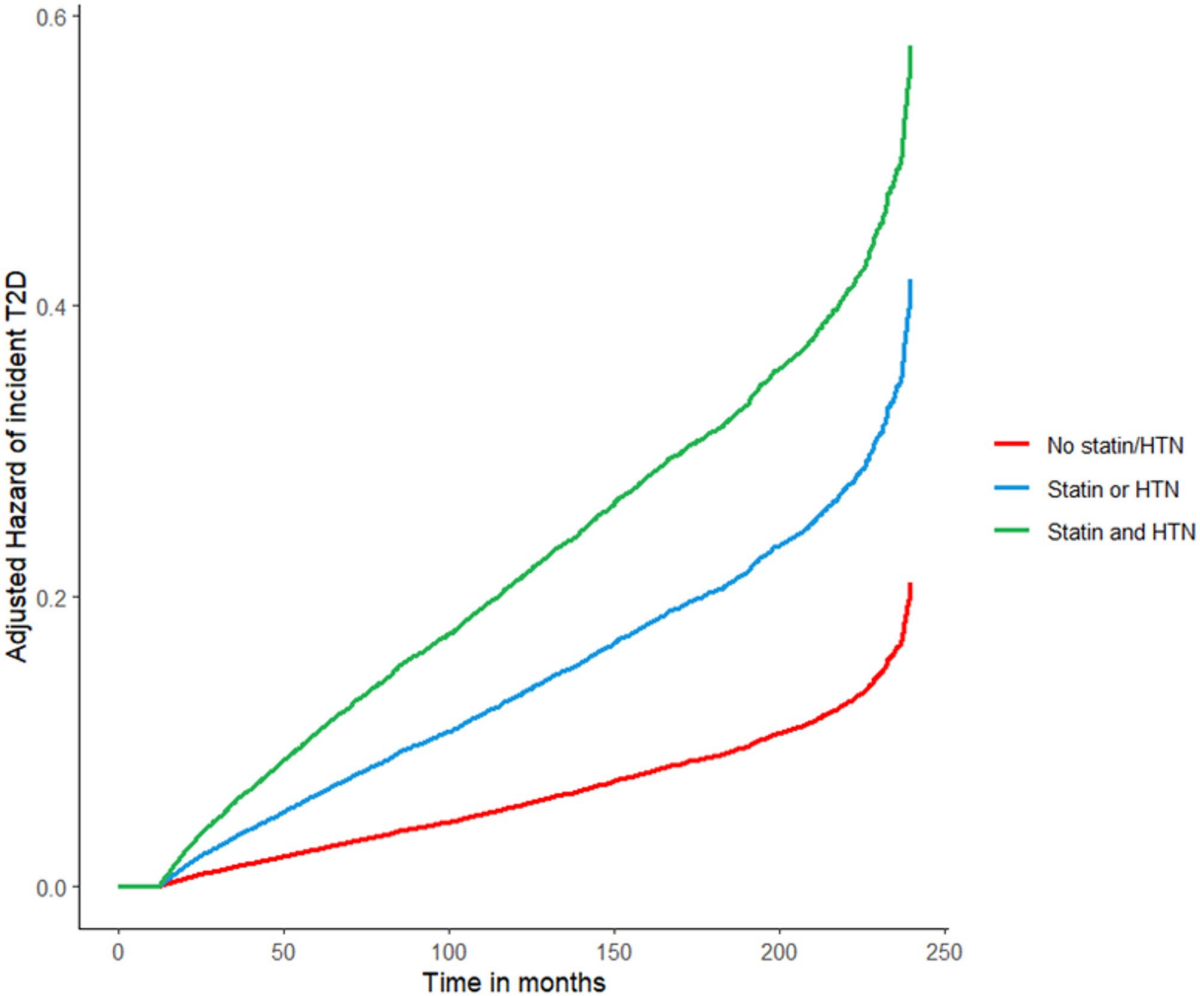
Adjusted hazard of incident T2D during the follow-up by the presence of arterial hypertension (HTN) and/or statin therapy or the absence of both

Table 3 shows the results of the Cox regression model obtained on the cohort adding among possible determinants of incident T2D the composite presence of statins therapy or hypertensive condition or of both statins and hypertension and as the reference the absence of both statin therapy and hypertension (complete cases *n* = 16446). This Cox regression showed that the presence of one between hypertension and statin therapy determines a 2-fold risk to develop incident T2D, while the combination of the two conditions together leads to a 4-fold higher risk (Fig. 3).

**Table 3:** Multiple Cox regression analysis performed to investigate variables associated with the risk of incident T2D over the observation period by the presence of Statins or hypertension or of both Statins and hypertension (the reference being the absence of both Statins therapy and hypertension)

Outcome incident T2D	aHR	95% CI	*p*-value
Age (years)	1.02	1.02, 1.02	< 0.001
Sex F vs. M	0.96	0.89, 1.05	0.403
Fasting plasma glucose (mg/dl)	1.03	1.03, 1.03	< 0.001
BMI (kg/m2)	1.04	1.03, 1.04	< 0.001
Serum creatinine (mg/dl)	1.40	1.20, 1.64	< 0.001
Total cholesterol (mg/dl)	0.99	0.99, 0.99	< 0.001
Statin/Hypertension			
No statin/No hypertension	–	–	
Statin therapy or hypertension	2.61	2.11, 3.22	< 0.001
Statin therapy and hypertension	4.62	3.75, 5.69	< 0.001

###  CV events

During the follow-up period, we recorded 1199 hard CV events (including 605 coronary and 594 cerebrovascular events). In particular, patients treated with statin therapy presented 260 and 274 coronary and cerebral fatal and non-fatal events, respectively, with a cumulative incidence of 1.9%. On the other hand, 665 fatal and non-fatal events were recorded in the control group, with an incidence of 0.7%. With a total of 1199 fatal and nonfatal CV events (0.6% of 119307 participants), our population does not allow a separate analysis for each component of the composite endpoint because of limited statistical power.

**Fig. 4:**
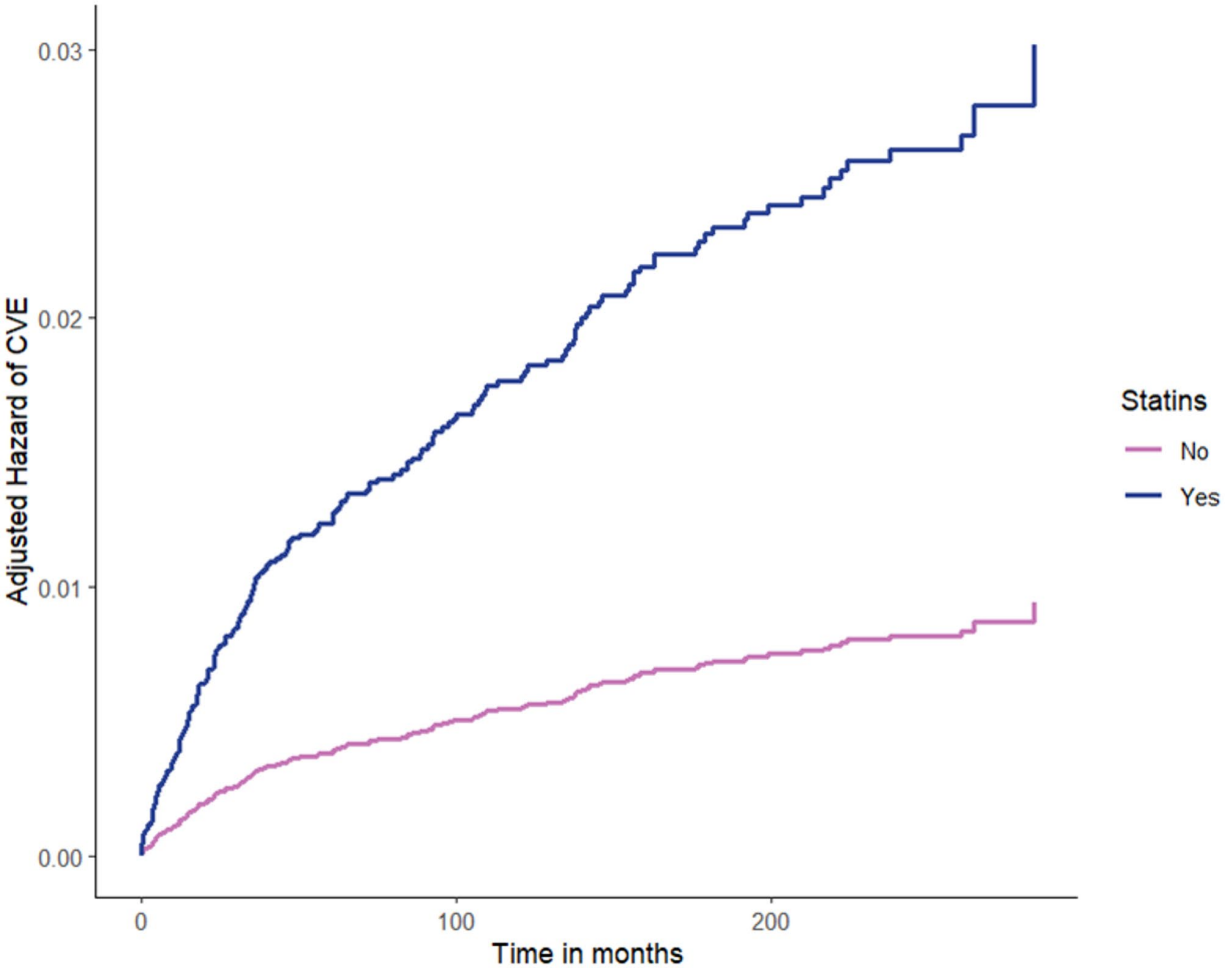
Adjusted hazard of cardiovascular events (CVE) during the follow-up by statin therapy

We then performed a Cox regression analysis to assess the effects of statins on CV events during follow-up, adjusting for age and sex, fasting plasma glucose, serum creatinine, total cholesterol, BMI, new-onset T2D, and hypertension (Table 4, complete cases *n* = 16446). Among these variables, age, serum creatinine and incident T2D resulted significantly associated with CV events. Indeed, this additional Cox regression analysis showed that the risk of CV events raised more rapidly (*p* < 0.001) in the group of patients treated with statins than in the controls, leading to a precocious separation of the curves of hazard of CV events of the two groups during the follow-up (Fig. 4).

**Table 4:** Multiple Cox regression analysis performed to investigate variables associated with the risk of CV events over the observation period

Outcome CV events	aHR	95% CI	*p*-value
Age (years)	1.06	1.04, 1.07	< 0.001
Sex F vs. M	1.31	0.98, 1.75	0.072
Fasting plasma glucose (mg/dl)	1.00	0.99, 1.00	0.311
Incident T2D	1.67	1.21, 2.30	0.002
BMI (kg/m^2^)	1.00	0.97, 1.03	0.902
Serum Creatinine (mg/dl)	1.72	1.03, 2.88	0.038
Statin therapy	3.27	2.11, 5.07	< 0.001
Hypertension	1.47	0.94, 2.30	0.087
Total cholesterol (mg/dl)	1.00	0.99, 1.00	0.090

**Fig. 5:**
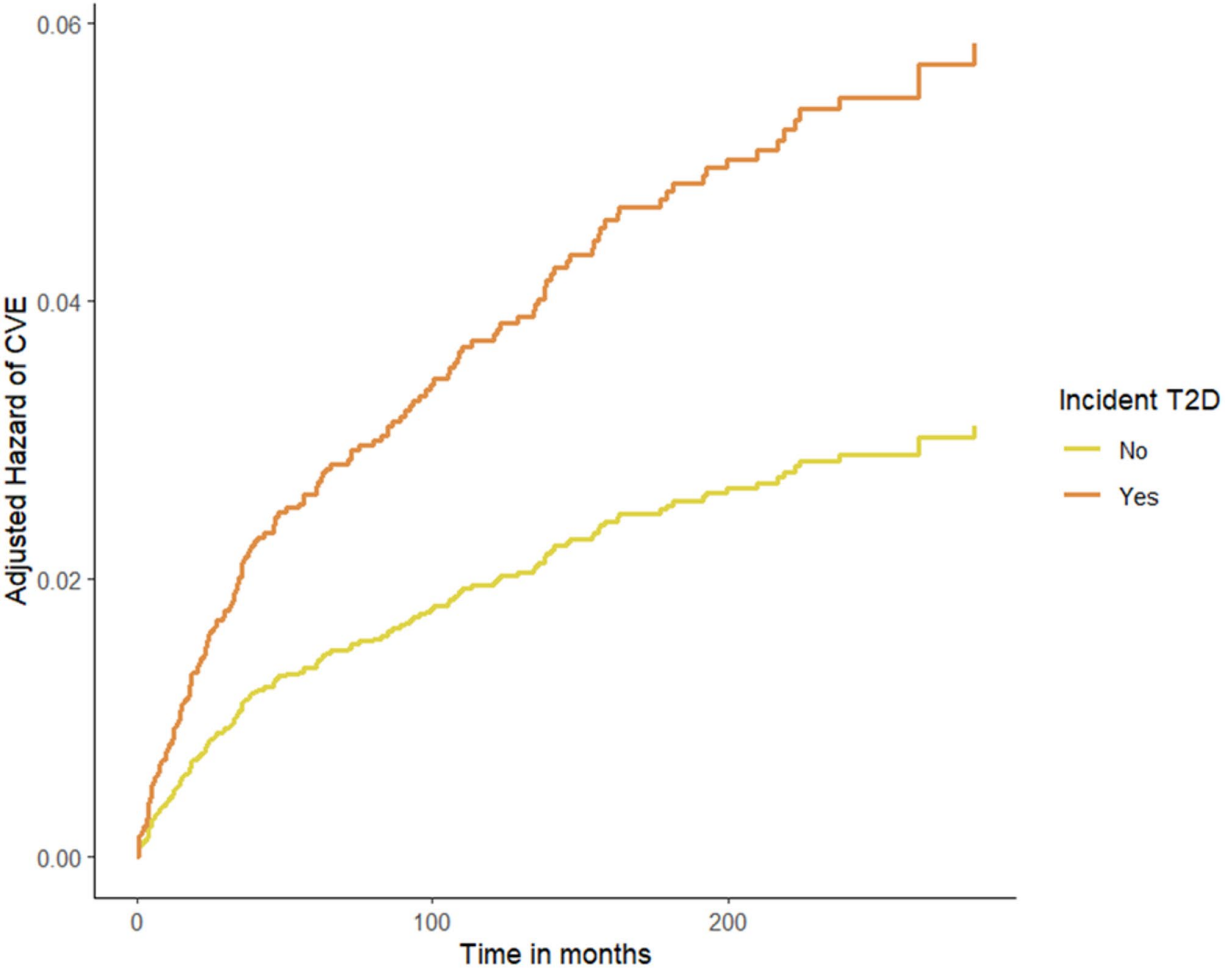
Adjusted hazard of CV events during the follow-up in the subgroup of statin treated individuals by incident T2D

Furthermore, in order to investigate the association between incident T2D and CV events, an adjusted Cox regression only in the subgroup of patients under statin therapy was performed (Table 5) and it demonstrated a significant association of CV events with age, serum creatinine levels and incident T2D. The two curves of the hazard risk for CV events rapidly diverge, highlighting the 40% increased risk to develop CV events in the group treated with statins and with incident T2D during the follow-up period (Fig. 5).

**Table 5:** Multiple Cox regression analysis performed only in the subgroup under Statin therapy to investigate variables associated with the risk of CV events over the observation period

Outcome CV events	aHR	95% CI	*p*-value
Age (years)	1.05	1.03, 1.07	< 0.001
Sex F vs. M	1.37	1.00, 1.87	0.047
Fasting plasma glucose (mg/dl)	1.00	0.99, 1.00	0.244
Incident T2D	1.92	1.38, 2.68	< 0.001
BMI (kg/m^2^)	1.00	0.96, 1.03	0.823
Serum creatinine (mg/dl)	1.88	1.10, 3.22	0.021
Hypertension	1.41	0.87, 2.27	0.164
Total cholesterol (mg/dl)	1.00	0.99, 1.00	0.234

### Propensity score matching (PSM)

Statin users in our real-world dataset differ substantially from non-users, as reported in Table 1: they are, on average, two decades older, with higher BMI, elevated fasting glucose levels, a threefold greater prevalence of hypertension, and increased cholesterol concentrations. We decided to match the 2 study groups for demographic and clinical factors using PSM. The baseline demographic characteristics of the population included in this analysis are shown in Supplementary Table 1. The absolute numbers of new diagnoses of T2D during the follow-up were 558 and 939 in the control and in the statin treated group, respectively. T2D incidence rate was 2.2% in the control group and 3.7% in the statin treatment group (*p* < 0.001). The HR for new-onset diabetes in statin treated patients was 1.72 (95% CI 1.55–1.91, *p* < 0.001) and the time course of the hazard of incident T2D during the follow-up was different (*p* < 0.001) between the two study groups (Supplementary Fig. 1). A separate model revealed that the presence of either hypertensive condition or statin therapy determines an HR to develop incident T2D of 2.35 (95% CI 1.84-3.00; *p* < 0.001), while the combination of the two conditions together leads to an HR of 4.79 (3.77,6.08; *p* < 0.001, Supplementary Fig. 2). Finally, the analysis performed to investigate the risk of CV events during the follow-up in patients with and without statin therapy showed an HR for statins of 3.46 (95% CI 2.20–5.44; *p* < 0.001, Supplementary Fig. 3).

## Discussion

The main findings of our study include: (1) statin treatment is associated with a higher incidence of new-onset T2D, even when used in primary prevention; (2) this effect is particularly marked in individuals with hypertension; (3) among statin treated patients, those who develop new T2D show an increased hazard of CV events; (4) the increased rate of incident T2D does not modify the class of CV risk in primary CV prevention.

To the best of our knowledge, our study is the first to be performed on a large sample of the general population and not on databases of RCT, hospitals, or outpatient clinics. Indeed, in Italy all citizens have a primary care physician (family doctor) who follows them even if they do not have specific pathologies. Therefore, our population includes not only patients with known illness but also individuals who do not have any disease (or at least are not aware of). This characteristic ensures the availability of a large population with low to moderate CV risk, which is difficult to attain in classic RCTs and hospital databases. Additionally, the exclusion of individuals with a prior diagnosis of T2D or ASCVD further reduced CV risk, as demonstrated by the low incidence of CV events in the global study population. In the statin-treated group, which also had a higher prevalence of common risk factors for new-onset T2D, a greater incidence of new T2D diagnoses was observed. Therefore, a multiple regression analysis was conducted to evaluate the independent role of statin therapy in this association. The results of this analysis demonstrate that statin use was associated with a 50% increased risk of developing T2D, with 12% of treated individuals receiving a T2D diagnosis after approximately six years of follow-up, a rate comparable to the ones reported in the CTT Collaboration meta-analysis [21] and in Lee’s meta-analysis [22] after high-intensity statin treatment over a shorter period in individuals with ASCVD.

Hypertension appears to have a similarly significant impact, as its presence is accompanied by a > 2-fold increase in the likelihood of new-onset T2D, aligning with observations of a higher incidence of diabetes in hypertensive patients [23]. Notably, statin therapy in hypertensive patients is associated with a risk of T2D that is more than fourfold than the one recorded in normotensive individuals not treated with statins.

The mechanisms underlying the effects of LDL-C lowering drug targets on fasting glucose are mostly unclear, but several potential pathophysiological pathways have been suggested [29–33]. For instance, HMGCR is the rate-limiting enzyme in the biosynthesis of cholesterol, and inhibiting the HMGCR pathway reduces the production of cholesterol [34]. Furthermore, inhibiting the HMGCR pathway decreases the synthesis of isoprenoid, thereby impairing cellular glucose uptake and inducing insulin resistance, which may explain the elevated blood glucose levels observed after the inhibition of the HMGCR pathway [35, 36]. This phenomenon may be particularly effective in hypertensive patients who already exhibit an impaired insulin sensitivity [37–39].

Finally, data on CV events further underscore the clinical significance of our findings. Albeit the incidence of CV events was twice as high in the statin-treated group compared to controls, its temporal pattern closely mirrored the one of new-onset T2D. Given that statin treatment per se does not directly cause CV events, this parallel trend raises the possibility of a causal link between T2D onset and increased CV risk. This hypothesis is further corroborated by the results of the propensity score matching. It is worth noting that, as we have previously shown [26], in the context of primary prevention, seven years of statin therapy—by maintaining LDL-C levels within the physiological range—can eliminate the excess CV risk associated with dyslipidemia. However, we do need to consider that the hazard of CV events of this population remains below the one indicated by the ESC guidelines for statin prescription [40], allowing us to consider that the increase in CV events associated with the rise in T2D induced by the use of statins in primary prevention should not discourage their utilization.

Our study is not exempt from limitations. We recognize that our findings do not permit any inference regarding the biological mechanisms responsible for the elevated incidence of new-onset T2D. We lack information on changes in lifestyle factors, including exercise and nutritional patterns, which might have contributed to the heightened T2D risk. Moreover, the extent of missing data for variables such as BMI, fasting plasma glucose, creatinine, and cholesterol does not permit dependable imputation, thereby constraining the sample size available for multivariable adjustments. A key strength of our investigation lies in the utilization of data derived from the general population. This strategy allowed us to address potential limitations related to unrecognized disease, which in RCTs may stem from limited post-randomization glycemic monitoring in individuals without previously diagnosed diabetes. Finally, the implementation of propensity score matching—encompassing nearly 17,000 individuals—resolves potential concerns on the fact that the elevated incidence of T2D among statin users could simply reflect inherent differences from non-users in observational datasets. The observation that findings from the propensity-matched cohort were entirely consistent with the overall results strongly reinforces our conclusions.

## Conclusion

The use of statin therapy in primary prevention is associated with an increased risk of incident T2D, particularly evident when statin therapy is combined with hypertension. Patients receiving statin therapy who develop incident T2D reveal an increased risk of CV events which, however, remains below the threshold required for prescription of statins according to the ESC guidelines, thus indicating that the observation of an increased risk of new-onset T2D does not modify the indications for the prescription of statins in primary prevention.

## Electronic supplementary material

Below is the link to the electronic supplementary material.

Supplementary Material 1

## Data Availability

No datasets were generated or analysed during the current study.
