# Correction: Statin-induced risk of diabetes does not reduce cardiovascular benefits in primary prevention: a 6-year propensity-score matched study in a large population

**DOI:** 10.1186/s12933-025-02834-1

**Published:** 2025-07-17

**Authors:** Maria Lembo, Valentina Trimarco, Raffaele Izzo, Daniela Pacella, Stanislovas S. Jankauskas, Paola Gallo, Roberto Piccinocchi, Carmine Morisco, Gaetano Piccinocchi, Luca Bardi, Stefano Cristiano, Giovanni Esposito, Giuseppe Giugliano, Fahimeh Varzideh, Maria Virginia Manzi, Bruno Trimarco, Gaetano Santulli

**Affiliations:** 1 Department of Advanced Biomedical Sciences, “Federico II” University, Naples, Italy; 2 Department of Neuroscience, Reproductive Sciences, and Dentistry, “Federico II” University, Naples, Italy; 3 Department of Public Health, “Federico II” University, Naples, Italy; 4 Department of Medicine, Einstein-Mount Sinai Diabetes Research Center (ES-DRC), Fleischer Institute for Diabetes and Metabolism (FIDAM), Wilf Family Cardiovascular Research Institute, Albert Einstein College of Medicine, New York City, NY USA; 5 “Luigi Vanvitelli” Hospital, Naples, Italy; 6 Academic Research Unit, International Translational Research and Medical Education (ITME) Consortium, Naples, Italy; 7 Italian Society for Cardiovascular Prevention (SIPREC), Rome, Italy; 8 COMEGEN Primary Care Physician Cooperative, Italian Society of General Medicine (SIMG), Naples, Italy; 9 Department of Molecular Pharmacology, Einstein Institute for Aging Research, Institute for Neuroimmunology and Inflammation (INI), Albert Einstein College of Medicine, New York City, NY USA

**Correction: Cardiovascular Diabetology (2025) 24:233** 10.1186/s12933-025-02798-2

In the original publication of this article [1], the typesetter has inadvertently missed the textual errors in Abstract, Methods and Results sections during the proof phase. The updated Abstract section and the textual errors have been corrected with this correction article. The original article has been corrected.

**Abstract**

**Background** The long-term risk of cardiovascular (CV) events in individuals who develop new-onset type 2 diabetes (T2D) after having received statin therapy in primary prevention is mostly unknown.

**Methods** We designed a population-based cohort study in individuals without T2D and atherosclerotic CV disease (ASCVD), divided in two groups according to the presence of statin therapy. We also balanced the study groups for demographic and clinical factors using propensity score matching.

**Results** 119307 individuals without T2D and ASCVD were divided in statin users (*N* = 90906) or not (*N* = 28401) and followed-up for 70.1 ± 61.3 months. Yearly incidence of T2D rate was 0.3% in the control group and 2.2% in the statin treated group. A Cox regression analysis confirmed the association between incident T2D and statin therapy. In normotensive individuals, the presence of statin therapy led to a twofold risk to develop incident T2D (HR: 2.61; 95% CI: 2.11–3.22, *p* < 0.001). In the hypertensive population, statin therapy was associated with a HR of incident T2D of 4.62 (95% CI: 3.75–5.69, *p* < 0.001). The rate of CV events including coronary and cerebrovascular fatal and non-fatal events, was 1.9% in the statin group vs. 0.7% in the control group and a multiple regression analysis demonstrated an association between statin therapy and CV events. A further Cox regression performed only in the statin treated population revealed a significant association of CV events with age, serum creatinine levels, and incident T2D. Of note, the increased rate of new-onset T2D associated with statin use does not modify the class of CV risk of this population. All these findings were confirmed by propensity score matched analysis.

**Conclusions** Statin therapy in primary prevention is associated with a higher risk of incident T2D, especially in hypertensive patients. However, since the final CV risk of those who develop T2D during statin treatment was lower than the one required for statin prescription according to the ESC guidelines, indicating that this phenomenon does not impair the benefit in CV prevention associated with the lipid lowering effect of statins.

In “Statistical analysis” under Methods section, the first sentence should read “Data were analyzed using IBM SPSS (version 29) and R statistical software version 4.4.0. Continuous variables mean ± standard deviation, while categorical variables are expressed as relative frequencies” instead of “Data were analyzed using IBM SPSS (version 29) and R statistical software version 4.4.0. Continuous variables are expressed as mean ± standard deviation, while categorical variables are expressed as relative frequencies”.

In Results section, the heading “Study participants and characteristics” was incorrectly given which should have been “Study participants”.

In the same section, the heading “Arterial hypertension” should follow Section 2 heading style.
