# Supplementary material for: Statin-induced risk of diabetes does not reduce cardiovascular benefits in primary prevention: a 6-year propensity-score matched study in a large population
Source: Cardiovasc Diabetol. 2025 May 31;24:233. doi: 10.1186/s12933-025-02798-2 (PMC12125874; doi:10.1186/s12933-025-02798-2)
Supplement: Supplementary file 1 — Supplementary Material 1 [file 12933_2025_2798_MOESM1_ESM.docx]

**Supplementary Table 1.** Baseline demographic characteristics of the 2 propensity score matched groups by statin therapy.

| **Variable** | **No statins N = 4,533** | **Statins N = 4,533** | **Difference (95% CI)** |
| --- | --- | --- | --- |
| **Age (years)** | 65 (12) | 66 (12) | -0.09 (-0.13, -0.05) |
| **Sex** |  |  | 0.02 (-0.02, 0.06) |
| **M** | 2,525 (56%) | 2,577 (57%) |  |
| **F** | 2,008 (44%) | 1,956 (43%) |  |
| **BMI (kg/m^2^)** | 26.7 (4.5) | 26.8 (4.3) | -0.02 (-0.06, 0.02) |
| **Fasting plasma glucose (mg/dl)** | 96 (17) | 97 (18) | -0.09 (-0.13, -0.05) |
| **Serum Creatinine (mg/dl)** | 0.88 (0.22) | 0.90 (0.24) | -0.07 (-0.11, -0.03) |
| **Hypertension (%)** | 2,902 (64%) | 2,976 (66%) | 0.02 (-0.04,0.01) |
| **Follow-Up (months)** | 90 (69) | 89 (68) | 0.02 (-0.02, 0.06) |
| **LDL cholesterol (mg/dl)** | 115 (28) | 114 (36) | -0.02 (-0.04, 0.07) |
| **Triglycerides (mg/dl)** | 104 (41) | 119 (45) | -0.35 (-0.40, -0.29) |

**Supplementary Figure 1.** Hazard of incident T2D during the follow up by statin therapy in the propensity score matched groups.

**
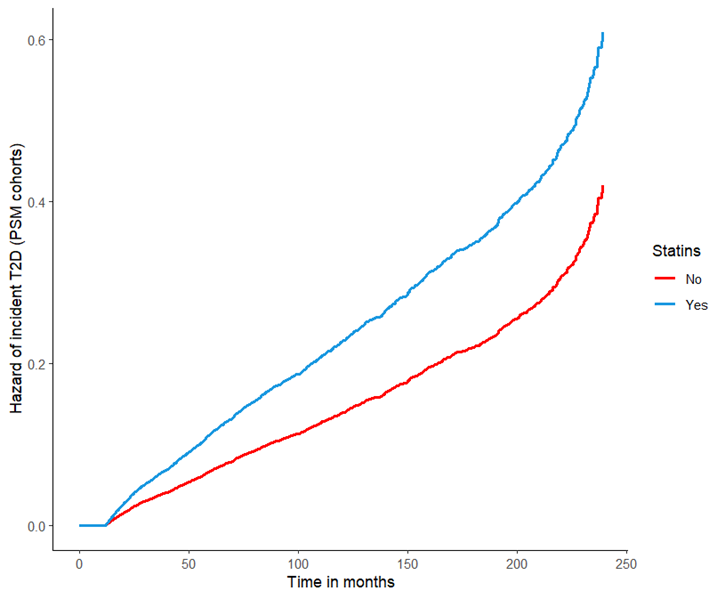
**

**Supplementary Figure 2.** Hazard of incident T2D during the follow up by the presence of arterial hypertension and/or statin therapy or the absence of both in the propensity score matched groups.

**
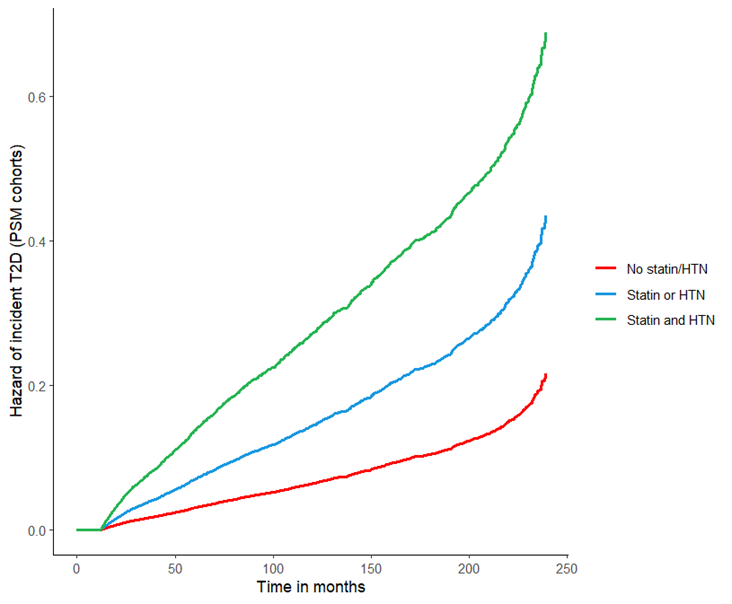
**

**Supplementary Figure 3.** Adjusted hazard of CVE during the follow up by statin therapy in the propensity score matched groups.

**
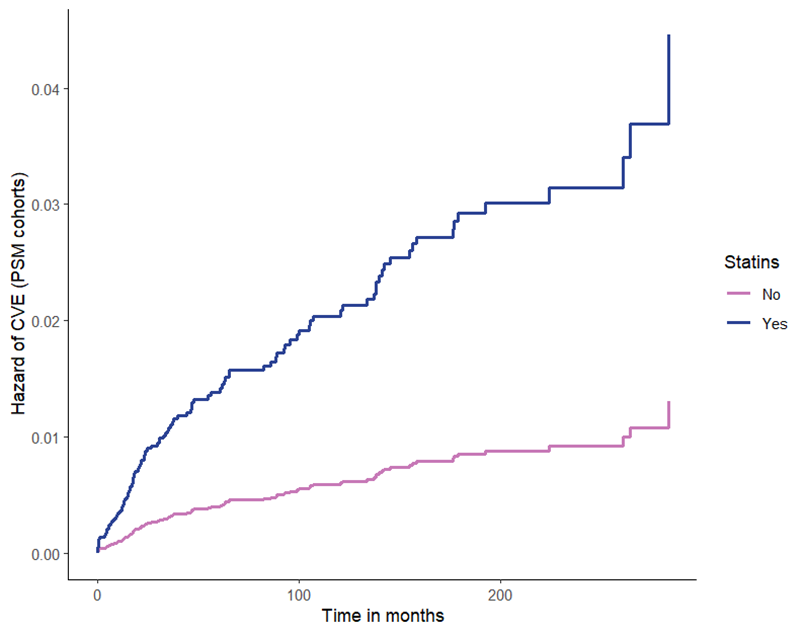
**
